# Necrotizing Soft Tissue Infection of the Breast during COVID-19 Pandemic

**DOI:** 10.1155/2020/8876475

**Published:** 2020-12-11

**Authors:** Nadia Kamagate, Robert DeVito

**Affiliations:** ^1^Western Reserve Health Education Trumbull Regional Medical Center, Department of Surgery, 1350 East Market Street Warren, Ohio, USA; ^2^Northeast Ohio Medical University, 4209 St. OH-44 Rootstown, Ohio 44272, USA

## Abstract

Necrotizing soft tissue infection has been historically recognized as a severe, rapidly spreading soft tissue infection associated with a very high risk of mortality. Cases of primary necrotizing fasciitis of the breast are rarely described but often fatal. We present a case of necrotizing soft tissue infection of the right breast extending to the anterior abdominal wall in a 39-year-old obese female, with a history of tobacco use. The patient presented 10 days after symptom onset due to concerns and anxiety related to COVID-19 exposure. This delay allowed for further extension and smoldering of the breast infection. The treatment of this aggressive disease process begins with early diagnosis, where a high index of suspicion is vital. Once diagnosed, the treatment regimen should be composed of emergent surgical debridement, which can include breast salvage debridement or total mastectomy, in addition to antibiotic therapy.

## 1. Introduction

Since the beginning of the coronavirus (COVID-19) pandemic in the US in March of 2020, many states including Ohio implemented orders to assist in limiting the spread of the virus and preservation of personal protective equipment (PPE). COVID-19 mandates included statewide stay-at-home orders and cancellation of nonessential or elective surgeries. Several studies have demonstrated that the coronavirus pandemic also led to rising anxiety and stress levels worldwide [1]. During this time, many emergency departments reported a startling decrease in the number of patients presenting with serious non-COVID-related medical issues. Due to fear of viral exposure, many patients choose to avoid seeking medical attention, resulting in delay of presentation and an associated increase in morbidity and mortality [2]. Additionally, as shown by Rubulotta et al., the challenges related to the sensitivity and specificity of testing for prompt diagnosis of the SARS-CoV-2 infection caused significant delays in recognizing and treating COVID-19 during the first wave of the pandemic [3].

Necrotizing soft tissue infections are considered an aggressively severe soft tissue infection associated with increased mortality. Historically, this infection mainly affects the abdominal wall, extremities, and perineum (commonly referred to as Fournier's Gangrene). Necrotizing soft tissue infections of the breast are quite rare, with very few cases reported in the literature. The key point in treating this disease process relies on early diagnosis and prompt surgical debridement, where any form of delay can have severe consequences.

## 2. Case

A 39-year-old female with a history of obesity and tobacco use presented to the emergency department with a 10-day history of increased swelling, redness, drainage, and pain of the right breast and upper abdomen. The patient reported associated fever, chills, nausea, and emesis. There was no history of trauma or previous surgery. She also stated she was reluctant to visit her primary care physician or any emergency department when her symptoms began due to anxiety related to COVID-19 viral exposure.

**Figure 1:**
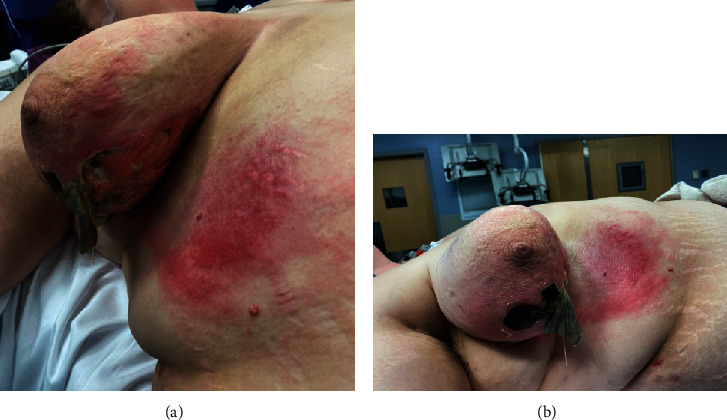
(a) Open right breast necrotic wound, with skin sloughing. (b) Erythema extending from the areolar complex to the superior aspect of the right anterior abdominal wall.

On clinical examination, the right breast was noted to have a 12 × 14 cm area of skin sloughing along the inferior inner aspect of the breast. A necrotic open wound was also seen within the lower outer quadrant with foul-smelling drainage surrounded by edema, erythema, and crepitus extending onto the right anterior abdominal wall (Figures 1(a) and 1(b)) The patient was noted to be hypotensive with mild tachycardia.

Pertinent laboratory findings revealed a leukocytosis of 16.9 × 10^9^/L with neutrophilia, thrombocytosis of 608 × 10^9^/L, and mild hyponatremia of 133 meq/L. Mild anemia was present, but renal function and lactic acid were found to be within normal limits.

**Figure 2:**
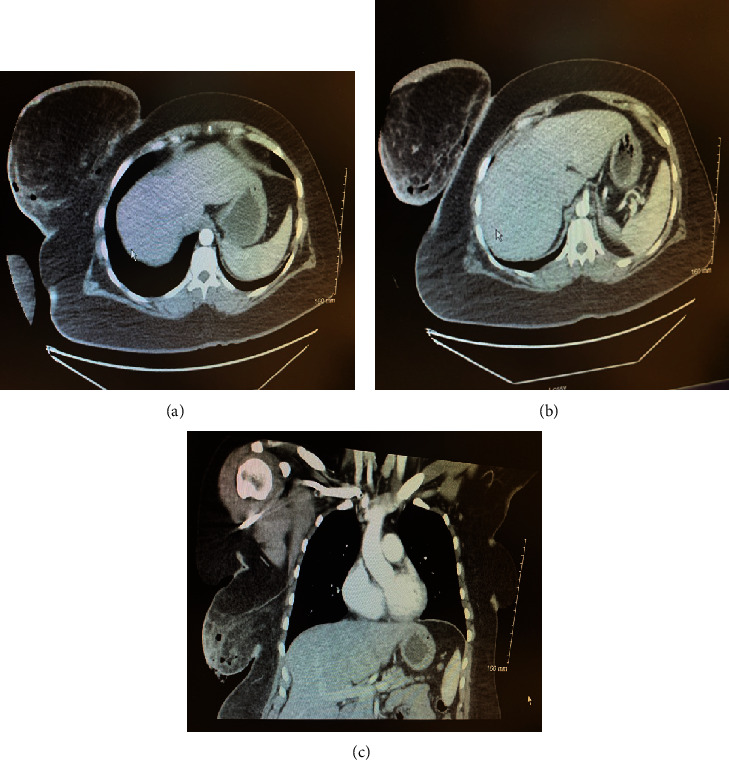
(a, b) Axial view of Chest CT depicting increased inflammation of the right breast and associated locules of gas along lateral and posterior aspect of breast. (c) Coronal view of Chest CT image showing increased attenuation of the right inferolateral breast tissue with several locules of gas. Images obtained with patient permission.

A chest computer tomography (CT) ordered by the emergency room physician revealed the right breast wound with several locules of gas within the superficial subcutaneous tissues of the right lateral and posterior breast with minimally increased attenuation of the subcutaneous tissues. (Figures 2(a)–2(c)) These findings were consistent with a necrotizing soft tissue infection, and general surgery was consulted. After surgical evaluation, a decision was made to proceed with emergent surgical intervention to provide source control. Prior to surgery, resuscitative intravenous fluids were administered in addition to vancomycin, clindamycin, and aztreonam, due to an unknown reported allergy to cephalexin.

The patient was taken to the operating room for an emergent wide excision and debridement of the infected tissue within the inferior right breast and the upper portion of the abdominal wall including the fascia overlying the pectoralis major muscle and external oblique. An attempt was made to obtain adequate source control while preserving as much breast tissue as possible, leaving the nipple areola complex intact. Cultures were obtained intraoperatively approximately 90 minutes after antibiotics had been administered. The wound was packed with wet-to-dry gauze and managed with daily dressing changes. An infectious disease consultation was performed, and the decision was made to replace aztreonam with piperacillin-tazobactam.

During the second operative debridement approximately 36 hours later, findings of advancing cellulitis extending superiorly on the right breast, encompassing the entire areolar complex, were identified. A completion mastectomy was performed in order to obtain adequate source control. Again, the wound was packed with planned daily local wound care and a return to the operating room for potential delayed primary closure. A third operation with minimal debridement of the lateral aspect of the right breast was performed and the wound, measuring 25 × 15 × 8 cm, underwent delayed primary closure in a multilayer fashion over closed suction drains.

**Figure 3:**
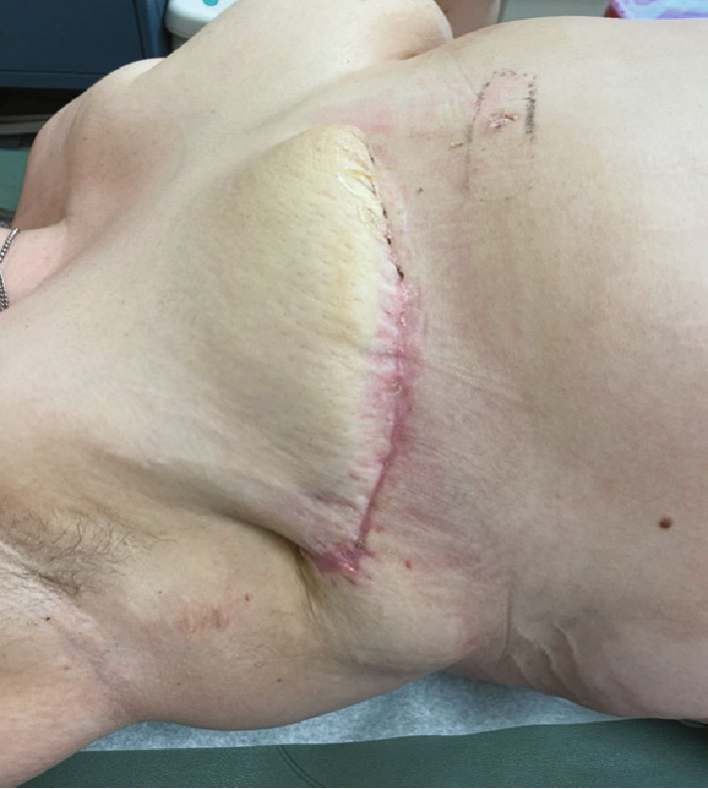
Well-healed mastectomy incision seen at 1-month postoperation; drains were removed.

Histopathologic evaluation of all tissue excised during all three operations was noted to be negative for malignancy and showed extensive acute suppurative inflammation, multiple abscesses, and tissue necrosis. The final tissue cultures grew Corynebacterium with negative blood cultures. The patient was discharged home 12 days after presentation on a 5-day course of ertapenem and daptomycin via a PICC line. Drains were removed during outpatient follow-up visit (Figure 3). The patient was offered plastic surgery consultation for possible reconstruction, but refused. The case underwent a multidisciplinary breast conference review two weeks after discharge, and no further recommendations were made.

## 3. Discussion

Necrotizing soft tissue infection (NSTI) is a rare severe disease characterized by a rapidly progressing infection causing fulminant tissue destruction with associated systemic toxicity. The aggressive nature of this disease process corresponds to its high mortality rate, which is 25-35% and has remained unchanged over the last 30 years. The high incidence of mortality in affected patients is directly proportional to the time to surgical intervention, where a delay in surgical debridement of 24 hours or more increased mortality to 70% [4]. In the US, the incidence of NSTI has been estimated to be 0.4 cases per 100,000 [5].

Characterization of NSTI has been based on anatomic location, depth of infection, and more commonly based on microbial source. When based on microbial sources, NSTI are classified as Type I, II, or III. Type I NSTI is typically due to a polymicrobial infection, including both aerobic and anaerobic microbes. These microbes include Group A beta-hemolytic *streptococcus*, *Staphylococcus aureus*, *Escherichia coli*, and *Clostridium* species. This is the most common class and is usually secondary to trauma or surgical insult. Type II NSTI consists of monomicrobial infections, with Group A beta-hemolytic *Streptococci* being the most common culprit. Type III NSTI is less commonly described as an infection caused by *Vibrio* vulnificus transmitted to humans by marine insects.

The pathophysiology of NSTI has been well established as an overwhelming subcutaneous infection with thrombosis of cutaneous perforating vessels resulting in necrosis. Common risk factors for the development of NSTI include trauma, burns, recent surgery, and comorbidities such as obesity, smoking, diabetes, peripheral vascular disease, liver disease, renal failure, and immunosuppression [4].

NSTI can occur anywhere in the body but commonly involves the lower extremities, perineum, and abdominal wall. Primary NSTI of the breast was first reported in 2001 by Shah et al. [6], and few cases have been described since then. Some cases have been described after breast surgical procedures, including simple and partial mastectomy, as well as core needle biopsies.

Within the breast, NSTI can be difficult to recognize and are commonly misdiagnosed as cellulitis, mastitis, abscess, or inflammatory breast cancer. This is thought to be secondary to the thickness of subcutaneous tissue between the skin and underlying deep fascia, in addition to the vast blood supply to the breast, which can both lead to delay in the presentation of common cutaneous changes [7]. Delays in diagnosis and or misdiagnosis both lead to delayed treatment and intervention, which can lead to fatal outcomes.

The Laboratory Risk Indicator for Necrotizing Fasciitis (LRINEC) scoring system is a useful adjunct that has been shown to aid in the early diagnosis of necrotizing soft tissue infections. The LRINEC score consists of six biologic markers: total white cell count, hemoglobin, sodium, glucose, serum creatinine, and C-reactive protein. A score of 6 or greater is associated with a positive predictor value of 92% [4]. This when used in conjunction with common clinical findings (including edema, erythema, localized pain, presence of fluid filled bullae, signs of hemodynamic instability) should trigger clinicians to initiate aggressive resuscitation with intravenous fluids and broad spectrum antibiotics, in addition to emergent surgical consultation for early operative debridement. Other diagnostic adjuncts include imaging studies such as MRI, ultrasound, chest-computed tomography noting marked tissue inflammation, and possible locules of gas within subcutaneous tissue.

Surgical wide debridement is the main treatment component of all NSTI, including that of the breast. Most previously reported cases in the literature reported single or staged surgical debridement(s), often time resulting in total mastectomy as a means of gaining infection control [8]. Some reports have described potential operative debridement with breast salvage in cases with early detection. In the setting of staged debridement, a second-look operation is typically planned 24-48 hours after index debridement to examine for further infection and or if the patient's clinical status worsens or fails to improve [9].

## 4. Conclusion

Although rare, necrotizing soft tissue infections can present in atypical locations such as the breast. Although disease recognition in the breast can be difficult, a high index of suspicion is needed to quickly diagnose and initiate early operative debridement (mastectomy vs. breast salvage debridement) and antimicrobial therapy. This multidisciplinary management approach is the mainstay in combating the high morbidity and mortality commonly associated with this disease process.

In addition, the healthcare issues brought on by the COVID-19 pandemic influence on the delay in presentation of patients resulting in conditions requiring more radical medical and/or surgical intervention. Thus, it is imperative that healthcare systems communicate with the public, the capacity and imperative need to handle all medical emergencies in a timely manner.
